# *trans*-K_3_[TcO_2_(CN)_4_]

**DOI:** 10.1107/S1600536810028205

**Published:** 2010-07-21

**Authors:** Sayandev Chatterjee, Andrew S. Del Negro, Matthew K. Edwards, Brendan Twamley, Jeanette A. Krause, Samuel A. Bryan

**Affiliations:** a Radiochemical Processing Laboratory, Pacific Northwest National Laboratory, Richland, WA 99357, USA; b Department of Chemistry, University of Idaho, Moscow, ID 83844, USA; c Department of Chemistry, University of Cincinnati, Cincinnati, OH 45221-0172, USA

## Abstract

The structure of the title compound, tripotassium *trans*-tetra­cyanidodioxidotechnetate(V), is isotypic with its Re analogue. The [TcO_2_(CN)_4_]^3−^ *trans*-tetra­cyanido­dioxido­technetate anion has a slightly distorted octa­hedral configuration. The Tc atom is located on a center of inversion and is bound to two O atoms in axial and to four cyanide ligands in equatorial positions. The Tc—O distance is consistent with a double-bond character. The two potassium cations, one located on a center of inversion and one in a general position, reside in octa­hedral or tetra­hedral environments, respectively. K⋯O and K⋯N inter­actions occur in the 2.7877 (19)–2.8598 (15) Å range.

## Related literature

The isotypic rhenate(V) analogue was reported by Fenn *et al.* (1971 ▶) (neutron study) and Murmann & Schlemper (1971 ▶) (X-ray study). For further information on dioxidotetra­cyanido anions of Tc and Re, see: Fackler *et al.* (1985 ▶); Kastner *et al.* (1982 ▶, 1984 ▶); Kremer *et al.* (1997 ▶). Luminescence properties of Tc complexes were reported by Del Negro *et al.* (2005 ▶, 2006 ▶). For further information on hydroxidooxidotetra­cyanido or aqua­oxidotetra­cyanido anions of Tc and Re, see: Baldas *et al.* (1990 ▶); Purcell *et al.* (1989 ▶, 1990 ▶). For general reviews on technetium structures, see: Bandoli *et al.* (2001 ▶, 2006 ▶); Bartholoma *et al.* (2010 ▶); Tisato *et al.* (1994 ▶). Synthetic details were given by Trop *et al.* (1980 ▶). For a description of the Cambridge Structural Database, see: Allen (2002 ▶).

## Experimental

### 

#### Crystal data

K_3_[TcO_2_(CN)_4_]
*M* *_r_* = 351.38
Triclinic, 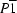

*a* = 6.2539 (6) Å
*b* = 6.9389 (6) Å
*c* = 7.4347 (7) Å
α = 108.305 (1)°
β = 109.816 (2)°
γ = 104.143 (1)°
*V* = 265.01 (4) Å^3^
*Z* = 1
Mo *K*α radiation
μ = 2.51 mm^−1^
*T* = 90 K
0.32 × 0.20 × 0.14 mm

#### Data collection

Bruker SMART APEX diffractometer
Absorption correction: multi-scan (*SADABS*; Sheldrick, 2007 ▶) *T*_min_ = 0.490, *T*_max_ = 0.705
3949 measured reflections
1097 independent reflections
1064 reflections with > σ(*I*)
*R*_int_ = 0.072

#### Refinement

*R*[*F* ^2^ > 2σ(*F* ^2^)] = 0.020
*wR*(*F* ^2^) = 0.049
*S* = 1.09
1097 reflections
68 parameters
Δρ_max_ = 0.77 e Å^−3^
Δρ_min_ = −0.90 e Å^−3^

### 

Data collection: *SMART* (Bruker, 2006 ▶); cell refinement: *SAINT* (Bruker, 2006 ▶); data reduction: *SAINT*; program(s) used to solve structure: *SHELXTL* (Sheldrick, 2008 ▶); program(s) used to refine structure: *SHELXTL*; molecular graphics: *SHELXTL* and *DIAMOND* (Brandenburg, 2010 ▶); software used to prepare material for publication: *SHELXTL*.

## Supplementary Material

Crystal structure: contains datablocks I, global. DOI: 10.1107/S1600536810028205/wm2375sup1.cif

Structure factors: contains datablocks I. DOI: 10.1107/S1600536810028205/wm2375Isup2.hkl

Additional supplementary materials:  crystallographic information; 3D view; checkCIF report

## Figures and Tables

**Table:** 

Tc1—O1	1.7721 (12)
Tc1—C1	2.1423 (19)
Tc1—C2	2.145 (2)
N1—C1	1.150 (3)
N2—C2	1.151 (3)

**Table:** 

N1—C1—Tc1	177.71 (16)
N2—C2—Tc1	172.74 (15)

**Fig. 1.**
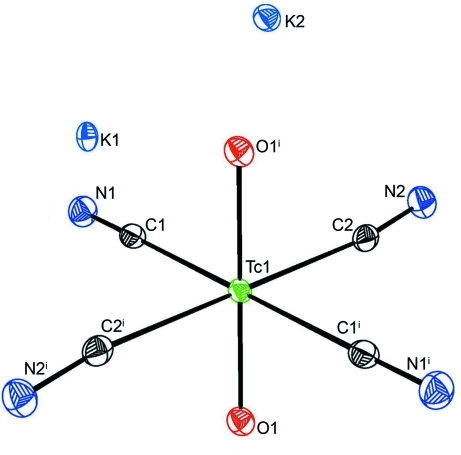
: The structural moieties of (I) with the atomic labelling scheme and 50% probability ellipsoids. [Symmetry code: (i) -x + 1, -y + 1, -z + 1].

**Fig. 2.**
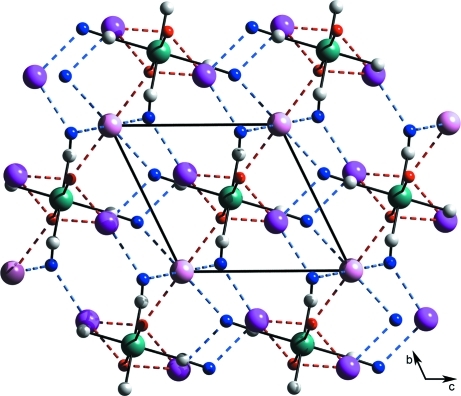
: The extended packing motif of (I) in the bc plane showing the octahedral and tetrahedral environments about the K+ ions. Dashed lines indicate the K···O (red) and K···N (blue) interionic interactions [K1 in light pink, K2 in dark pink].

## supplementary crystallographic information

### Comment

^99^Tc is the most significant long-lived product of uranium fission. In addition to a long half-life (2.13x10^5^ yrs), it is readily water soluble, making it extremely mobile in the environment. This, coupled with its ability to form anionic species, causes major concern when considering long-term disposal of high-level radioactive waste. Thus, it is imperative to provide methods for chemical detection of ^99^Tc. Under normal environmental conditions, ^99^Tc composition is dominated by the pertechnetate anion (TcO_4_^-^) which lacks a characteristic spectral signature. This prevents its rapid, sensitive and economic *in situ* detection. In order to address this problem, our research is focused on designing a suitable sensor for the detection of TcO_4_^-^. The ultimate aim is to chemically convert the pertechnetate anion to an organic-ligated species that will have a readily characterizable spectral signature. As a first step to address this problem, our group has been able to identify several technetium complexes with long-lived excited states. Thus, the luminescence properties of technetium(V)-dioxidotetrapyridyl and technetium(II)-tris(1,2-bis(dimethylphosphino)ethane) were reported by Del Negro *et al.* (2005, 2006).

However, the significant dearth of Tc structures in general (Bandoli *et al.*, 2001, 2006; Bartholoma *et al.*, 2010; Tisato *et al.*, 1994) has resulted in a substantial knowledge gap in the structures and bonding of technetium complexes, thereby preventing the correlation of the electronic properties with structural parameters. As a representative comparison, the Cambridge Structural Database (CSD, version 5.31; Allen, 2002) currently contains 21 dioxidotechnetium complexes compared to 141 structures reported for dioxidorhenium and dioxidomanganese complexes. In an attempt to bridge this gap, we are focusing on the structural characterization of a series of dioxidotechnetium(V) complexes. Herein, we report the structure of K_3_[TcO_2_(CN)_4_], (I), a tetracyanidodioxidotechnetium(V) salt.

The unit cell of (I) is comprised of three K^+^ cations and one discrete [TcO_2_(CN)_4_]^3-^ anion. The configuration of the anion is shown in Fig. 1. The Tc atom (located on an inversion center) resides in an octahedral environment defined by two oxido and four cyanido ligands. The *trans* basal C—Tc—C angles are linear resulting in a square planar arrangement of the tetracyanido groups about the Tc center.

The Tc═O distance (1.7721 (12) Å) is virtually identical to the Re═O distance in K_3_[ReO_2_(CN)_4_] [neutron study: 1.773 (8) Å (Fenn *et al.*, 1971), X-ray study: 1.781 (3) Å (Murmann & Schlemper, 1971)]. An average Tc═O distance of 1.74 Å has been observed for an oxido ligand in cations of the type *trans*-[O_2_*R*_4_Tc]^+^ (*R* = 4-*tert*-butylpyridine, imidazole, 1-methylimidazole, trimethylenediamine) (Kastner *et al.*, 1984; Fackler *et al.*, 1985; Kremer *et al.*, 1997) or *trans*-[O_2_en_2_Tc]^+^ (en = ethylenediamine) (Kastner *et al.*, 1982) while a Tc—O distance of 2.559 (9) Å is observed in the [TcN(CN)_4_(OH_2_)]^2-^ dianion reported by Baldas *et al.* (1990).

The Tc—C distances (2.1423 (19) and 2.145 (2) Å) determined in this study are slightly longer than reported by Murmann & Schlemper (1971) and Fenn *et al.* (1971) for the [ReO_2_(CN)_4_]^3-^ anion (Re—C_ave_ = 2.13 Å). In addition, we see a deviation from linearity for one Tc—C—N angle (172.74 (15)° *versus* 177.71 (16)°). Similar bending is common in the [ReO_2_(CN)_4_]^3-^, [ReO(OH)(CN)_4_]^2-^ and [ReO(OH_2_)(CN)_4_]^-^ anions (ave. 175° *versus* 178°) (Fenn *et al.*, 1971; Murmann & Schlemper, 1971; Purcell *et al.*, 1989, 1990) and may be a result of inequivalent interactions with the surrounding environment.

(I) shows interionic interactions between the K^+^ cations and the [TcO_2_(CN)_4_]^3-^ anion. The K^+^ cations reside in two distinct environments [K1 located on a special position with inversion symmetry and K2 is located on a general position] (Fig 2). Each oxygen atom of the anion interacts with one K1 and two K2 atoms. The local environment about K1 is distorted octahedral, consisting of the following interactions: K1···O1 = 2.8235 (13) Å, K1···N1 = 2.8315 (18) Å, K1···N2 = 2.8598 (15) Å and the symmetry equivalents. On the other hand, the local environment about K2 is approximately tetrahedral with interactions of K2···O1 = 2.7936 (12) and 2.8262 (14) Å, K2···N1 = 2.8152 (16) Å and K2···N2 = 2.7877 (19) Å). In addition, there are three significantly longer contacts between K2 and N1 (3.1710 (16) and 3.5715 (16) Å) and K2 and N2 (3.1496 (17) Å).

### Experimental

(I) is prepared from [TcO_2_(py)_4_]Cl (py = pyridyl) by the method of Trop *et al.* (1980). Complete cyanide substitution of the pyridyl groups of the starting material is achieved by adding an excess of alkaline cyanide.

In a typical preparation, 0.5 g of [TcO_2_(py)_4_]Cl was dissolved in a minimum amount of methanol. Addition of 50 ml of 1.2*M* KCN in 5:1 (*v*/*v*) methanol/water to the above resulted in an immediate green solution which turned yellow in approx. 5 minutes. This was followed by a gradual appearance of a fine yellow precipitate. The mixture was stirred on a hot plate set to low heat for an additional hour to ensure complete conversion. The supernatant was drawn off and the yellow precipitate was washed with diethyl ether. The yellow precipitate was dissolved in a minimal amount of water, followed by slow diffusion of methanol vapors, to yield crystals of (I) suitable for X-ray diffraction.

CAUTION! All syntheses and characterizations were performed with ^99^Tc, which is a β-emitting isotope with a half-life of 2.13x10^5^ years. The handling of small quantities (generally <100 milligrams) of this material does not pose a serious health hazard since common laboratory materials provide adequate shielding. However, radiation safety procedures must be used to prevent contamination!

### Crystal data

**Table:**

K3[TcO_2_(CN)_4_]	*Z* = 1
*M**_r_* = 351.38	*F*(000) = 168
Triclinic, *P*1	*D*_x_ = 2.202 Mg m^−^^3^
Hall symbol: -P 1	Mo *K*α radiation, λ = 0.71073 Å
*a* = 6.2539 (6) Å	Cell parameters from 3550 reflections
*b* = 6.9389 (6) Å	θ = 3.2–30.0°
*c* = 7.4347 (7) Å	µ = 2.51 mm^−^^1^
α = 108.305 (1)°	*T* = 90 K
β = 109.816 (2)°	Fragment, yellow
γ = 104.143 (1)°	0.32 × 0.20 × 0.14 mm
*V* = 265.01 (4) Å^3^	

### Data collection

**Table:**

Bruker SMART APEX diffractometer	1097 independent reflections
Radiation source: normal-focus sealed tube	1064 reflections with > σ(*I*)
graphite	*R*_int_ = 0.072
ω scans	θ_max_ = 26.5°, θ_min_ = 3.2°
Absorption correction: multi-scan (*SADABS*; Sheldrick, 2007)	*h* = −7→7
*T*_min_ = 0.490, *T*_max_ = 0.705	*k* = −8→8
3949 measured reflections	*l* = −9→9

### Refinement

**Table:**

Refinement on *F*^2^	Primary atom site location: structure-invariant direct methods
Least-squares matrix: full	Secondary atom site location: difference Fourier map
*R*[*F*^2^ > 2σ(*F*^2^)] = 0.020	*w* = 1/[σ^2^(*F*_o_^2^) + (0.0114*P*)^2^ + 0.0622*P*] where *P* = (*F*_o_^2^ + 2*F*_c_^2^)/3
*wR*(*F*^2^) = 0.049	(Δ/σ)_max_ = 0.001
*S* = 1.09	Δρ_max_ = 0.77 e Å^−^^3^
1097 reflections	Δρ_min_ = −0.90 e Å^−^^3^
68 parameters	Extinction correction: *SHELXTL* (Sheldrick, 2008), Fc^*^=kFc[1+0.001xFc^2^λ^3^/sin(2θ)]^-1/4^
0 restraints	Extinction coefficient: 0.024 (3)

### Special details

**Table:**

Experimental. A suitable crystal was mounted on a glass fiber and immediately transferred to the goniostat bathed in a cold stream.CAUTION!All syntheses and characterizations were performed with^99^Tc, which is a β-emitting isotope with a half-life of 2.13x10^5^ years. The handling of small quantities (generally <100 milligrams) of this material does not pose a serious health hazard since common laboratory materials provide adequate shielding. However, radiation safety procedures must be used to prevent contamination.
Geometry. All e.s.d.'s (except the e.s.d. in the dihedral angle between two l.s. planes) are estimated using the full covariance matrix. The cell e.s.d.'s are taken into account individually in the estimation of e.s.d.'s in distances, angles and torsion angles; correlations between e.s.d.'s in cell parameters are only used when they are defined by crystal symmetry. An approximate (isotropic) treatment of cell e.s.d.'s is used for estimating e.s.d.'s involving l.s. planes.
Refinement. Refinement of *F*^2^ against ALL reflections. The weighted *R*-factor *wR* and goodness of fit *S* are based on *F*^2^, conventional *R*-factors *R* are based on *F*, with *F* set to zero for negative *F*^2^. The threshold expression of *F*^2^ > σ(*F*^2^) is used only for calculating *R*-factors(gt) *etc*. and is not relevant to the choice of reflections for refinement. *R*-factors based on *F*^2^ are statistically about twice as large as those based on *F*, and *R*-factors based on ALL data will be even larger.

### Fractional atomic coordinates and isotropic or equivalent isotropic displacement parameters (Å^2^)

**Table:**

	*x*	*y*	*z*	*U*_iso_*/*U*_eq_	
K1	0.5000	0.0000	1.0000	0.01358 (15)	
K2	0.04367 (6)	0.33536 (7)	0.69923 (6)	0.01283 (13)	
Tc1	0.5000	0.5000	0.5000	0.00885 (11)	
O1	0.6628 (2)	0.3641 (2)	0.39016 (18)	0.0123 (3)	
N1	0.6522 (3)	0.3389 (3)	0.8804 (2)	0.0159 (4)	
N2	−0.0098 (3)	0.0509 (3)	0.2506 (2)	0.0161 (4)	
C1	0.5999 (3)	0.3996 (3)	0.7507 (3)	0.0120 (4)	
C2	0.1707 (3)	0.2034 (3)	0.3254 (3)	0.0123 (4)	

### Atomic displacement parameters (Å^2^)

**Table:**

	*U*^11^	*U*^22^	*U*^33^	*U*^12^	*U*^13^	*U*^23^
K1	0.0127 (3)	0.0130 (3)	0.0118 (3)	0.0033 (2)	0.0024 (2)	0.0064 (2)
K2	0.0118 (2)	0.0155 (3)	0.0129 (2)	0.00591 (17)	0.00588 (15)	0.00746 (18)
Tc1	0.00763 (14)	0.01073 (17)	0.00889 (14)	0.00383 (10)	0.00354 (9)	0.00513 (10)
O1	0.0110 (6)	0.0132 (7)	0.0131 (6)	0.0052 (5)	0.0050 (5)	0.0063 (5)
N1	0.0157 (7)	0.0175 (10)	0.0145 (7)	0.0059 (7)	0.0061 (6)	0.0084 (7)
N2	0.0139 (7)	0.0172 (9)	0.0164 (7)	0.0056 (7)	0.0055 (6)	0.0085 (7)
C1	0.0100 (8)	0.0120 (10)	0.0121 (8)	0.0033 (7)	0.0055 (6)	0.0035 (7)
C2	0.0136 (8)	0.0157 (11)	0.0106 (8)	0.0084 (8)	0.0055 (6)	0.0071 (7)

### Geometric parameters (Å, °)

**Table:**

Tc1—O1^i^	1.7721 (12)	N2—K2^v^	2.7876 (19)
Tc1—O1	1.7721 (12)	N2—K1^vi^	2.8598 (15)
Tc1—C1^i^	2.1423 (19)	K1—O1^vii^	2.8235 (13)
Tc1—C1	2.1423 (19)	K1—O1^viii^	2.8235 (13)
Tc1—C2	2.145 (2)	K1—N1	2.8315 (18)
Tc1—C2^i^	2.145 (2)	K1—N1^ix^	2.8315 (18)
O1—K2^ii^	2.7937 (12)	K1—N2^x^	2.8598 (15)
O1—K1^iii^	2.8235 (13)	K1—N2^v^	2.8598 (15)
O1—K2^i^	2.8262 (14)	K2—N2^v^	2.7877 (19)
N1—C1	1.150 (3)	K2—O1^xi^	2.7936 (12)
N1—K2^iv^	2.8152 (16)	K2—N1^iv^	2.8152 (16)
N1—K2^ii^	3.1710 (16)	K2—O1^i^	2.8262 (14)
N2—C2	1.151 (3)	K2—N1^xi^	3.1710 (16)
			
O1^i^—Tc1—O1	180.0	C2—N2—K2^v^	124.85 (13)
O1^i^—Tc1—C1^i^	90.13 (6)	C2—N2—K1^vi^	127.59 (15)
O1—Tc1—C1^i^	89.87 (6)	K2^v^—N2—K1^vi^	107.51 (6)
O1^i^—Tc1—C1	89.87 (6)	O1^vii^—K1—O1^viii^	180.00 (5)
O1—Tc1—C1	90.13 (6)	O1^vii^—K1—N1	97.86 (4)
C1^i^—Tc1—C1	179.999 (1)	O1^viii^—K1—N1	82.14 (4)
O1^i^—Tc1—C2	88.60 (6)	O1^vii^—K1—N1^ix^	82.14 (4)
O1—Tc1—C2	91.40 (6)	O1^viii^—K1—N1^ix^	97.86 (4)
C1^i^—Tc1—C2	92.34 (7)	N1—K1—N1^ix^	180.00 (7)
C1—Tc1—C2	87.66 (7)	O1^vii^—K1—N2^x^	104.18 (4)
O1^i^—Tc1—C2^i^	91.40 (6)	O1^viii^—K1—N2^x^	75.82 (4)
O1—Tc1—C2^i^	88.60 (6)	N1—K1—N2^x^	95.00 (5)
C1^i^—Tc1—C2^i^	87.66 (7)	N1^ix^—K1—N2^x^	85.00 (5)
C1—Tc1—C2^i^	92.34 (7)	O1^vii^—K1—N2^v^	75.82 (4)
C2—Tc1—C2^i^	180.0	O1^viii^—K1—N2^v^	104.18 (4)
Tc1—O1—K2^ii^	111.70 (5)	N1—K1—N2^v^	85.00 (5)
Tc1—O1—K1^iii^	131.58 (5)	N1^ix^—K1—N2^v^	95.00 (5)
K2^ii^—O1—K1^iii^	107.62 (4)	N2^x^—K1—N2^v^	180.0
Tc1—O1—K2^i^	106.54 (6)	N2^v^—K2—O1^xi^	123.16 (4)
K2^ii^—O1—K2^i^	98.26 (4)	N2^v^—K2—N1^iv^	101.64 (5)
K1^iii^—O1—K2^i^	94.38 (4)	O1^xi^—K2—N1^iv^	125.77 (5)
N1—C1—Tc1	177.71 (16)	N2^v^—K2—O1^i^	139.59 (4)
N2—C2—Tc1	172.74 (15)	O1^xi^—K2—O1^i^	81.74 (4)
C1—N1—K2^iv^	116.78 (14)	N1^iv^—K2—O1^i^	82.38 (5)
C1—N1—K1	145.03 (14)	N2^v^—K2—N1^xi^	84.21 (5)
K2^iv^—N1—K1	94.44 (5)	O1^xi^—K2—N1^xi^	77.02 (4)
C1—N1—K2^ii^	73.15 (11)	N1^iv^—K2—N1^xi^	79.06 (5)
K2^iv^—N1—K2^ii^	100.94 (5)	O1^i^—K2—N1^xi^	135.31 (4)
K1—N1—K2^ii^	118.11 (6)		

Symmetry codes: (i) −*x*+1, −*y*+1, −*z*+1; (ii) *x*+1, *y*, *z*; (iii) *x*, *y*, *z*−1; (iv) −*x*+1, −*y*+1, −*z*+2; (v) −*x*, −*y*, −*z*+1; (vi) *x*−1, *y*, *z*−1; (vii) −*x*+1, −*y*, −*z*+1; (viii) *x*, *y*, *z*+1; (ix) −*x*+1, −*y*, −*z*+2; (x) *x*+1, *y*, *z*+1; (xi) *x*−1, *y*, *z*.
